# Characterization of sub-micrometre-sized voids in fixed human brain tissue using scanning X-ray microdiffraction

**DOI:** 10.1107/S1600576724008987

**Published:** 2024-10-01

**Authors:** Prakash Nepal, Abdullah A. Bashit, Lee Makowski

**Affiliations:** a Department of Bioengineering Northeastern University Boston MA02115 USA; b Department of Electrical and Computer Engineering Northeastern University Boston MA02115 USA; c Department of Chemistry and Chemical Biology Northeastern University Boston MA02115 USA; Australian Centre for Neutron Scattering, ANSTO, Australia

**Keywords:** X-ray microdiffraction, small-angle X-ray scattering, SAXS, wide-angle X-ray scattering, WAXS, *in situ* structural studies, neurodegenerative diseases

## Abstract

Using a 5 µm-diameter X-ray beam, we collected scanning X-ray microdiffraction in both the small-angle (SAXS) and the wide-angle (WAXS) regimes from thin sections of fixed human brain tissue from Alzheimer’s subjects. The intensity of scattering in the SAXS regime of these patterns exhibits essentially no correlation with the observed intensity in the WAXS regime, indicating that the structures responsible for these two portions of the diffraction patterns, which reflect different length scales, are distinct. SAXS scattering exhibits a power-law behavior in which the log of intensity decreases linearly with the log of the scattering angle. The slope of the log–log curve is roughly proportional to the intensity in the SAXS regime and, surprisingly, inversely proportional to the intensity in the WAXS regime. We interpret these observations as being due to the presence of sub-micrometre-sized voids formed during dehydration of the fixed tissue. The SAXS intensity is due largely to scattering from these voids, while the WAXS intensity derives from the secondary structures of macromolecular material surrounding the voids. The ability to detect and map the presence of voids within thin sections of fixed tissue has the potential to provide novel information on the degradation of human brain tissue in neurodegenerative diseases.

## Abstract 2

Using scanning X-ray microdiffraction, we discovered that the small-angle scattering from human brain tissue is due largely to the presence of sub-micrometre-sized voids formed during dehydration of the fixed tissue, while the intensity at wide angles derives from the macromolecular material surrounding the voids. The ability to detect and map the presence of voids within thin sections of fixed tissue has the potential to provide novel information on the degradation of human brain tissue in neurodegenerative disease.

## 1. Introduction

The intrinsic heterogeneity of brain tissue makes its study using scattering techniques challenging. However, the availability of micro- and nano-scale X-ray beams at synchrotron sources enables the measurement of scattering from small volumes that may be predominantly composed of a single constituent, thereby resulting in data that are potentially interpretable in terms of the structure of that constituent (Liu & Makowski, 2022 ▸). Furthermore, the collection of thousands of diffraction patterns from scans of tissue thin sections makes possible recognition of correlations between patterns and between distinct parts of the patterns in ways that would be impossible using individual exposures. This capability represents a powerful tool for the characterization of heterogeneous materials.

Here we present an analysis of the properties of small- and wide-angle X-ray scattering (SAXS and WAXS) from histological sections of fixed human brain tissue and outline strategies for extracting information from these data. Sample preparation alters the properties of these sections in well characterized ways. The tissue is initially embedded in paraffin, sectioned and then de-paraffinized, a process which involves washing with xylene and then ethanol. This removes much of the lipid (and paraffin) and dehydrates the material (Werner *et al.*, 2000 ▸). The protein tertiary structure may be disrupted in fixed tissue, but the secondary structure remains largely intact. Most epitopes appear to be preserved, and the overall spatial distribution of constituents remains unchanged. The fibrillar cross-β aggregates associated with Alzheimer’s and other neurogenerative diseases are particularly resilient to the physical and chemical processes involved in standard histological preparation (Zohdi *et al.*, 2015 ▸), and formalin fixation immobilizes these fibrils within the cross-linked matrix of tissue (Liu *et al.*, 2016 ▸). Although the arrangement of molecular constituents may be altered by this process, the properties of the samples remain of intrinsic interest for what they may reveal of the original, unfixed tissue once the impact of tissue preparation is considered.

The scattering from the often randomly oriented cross-β fibrils embedded in the matrix of fixed tissue has many of the properties of scattering from fibrils in aqueous solution (Roig-Solvas & Makowski, 2017 ▸; Nepal *et al.*, 2022 ▸). The analysis of the SAXS scattering from fibrils is complicated by the presence of the tissue matrix in which the fibrils are embedded, resulting in small-angle interference that gives rise to a non-linear Guinier plot. As will be shown here, the predicted cross-terms between fibrils and tissue matrix (Nepal *et al.*, 2022 ▸) are further obscured by scattering from voids, most probably formed during dehydration of the tissue. The electron density contrast between void and tissue is much greater than that between fibril and tissue and thereby gives rise to intense scattering (Nepal & Saldin, 2018 ▸) that dominates the small-angle part of these patterns.

In the small-angle regime, scattering from all tissue samples examined exhibited, over a limited *q* range, a power-law behavior with
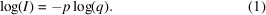
Here, *I* is the intensity and *q* is the momentum transfer [*q* = 4π sin(θ)/λ, where 2θ is the scattering angle and λ is the X-ray wavelength] and *p* is the negative of the slope of the log–log curve, referred to here as the ‘scattering exponent’. In most cases, this linear behavior was observed from *q* ≃ 0.0075 Å^−1^ to *q* ≃ 0.07 Å^−1^ (where *q* = 0.005 Å^−1^ is the smallest *q* for which intensity was observed in these experiments).

Power-law behavior is commonly observed in SAXS and, because the exponent *p* has often been interpreted in terms of fractal geometry (Mandelbrot, 1983 ▸), could be related to structural features that include surface fractals and diffused interfaces in two-phase systems; monodisperse dimensional objects including rods, discs or Gaussian polymer coils; and poly-dispersed particles. Materials that give rise to (negative) slopes of between 3 and 4 (as we report here) are frequently described in terms of surface fractals (Avnir *et al.*, 1984 ▸; Pfeifer *et al.*, 1983 ▸). However, we demonstrate that the power-law behavior of equation (1) may be attributed to a heterogeneous population of sub-micrometre voids with a scale-free size distribution within the scattering material.

We find a lack of correlation between the intensities in the SAXS and WAXS regimes which was unanticipated and might have continued to be unnoticed except for the power of the SAXS/WAXS scanning microdiffraction to produce thousands of scattering patterns. Evidence for the observed SAXS intensities being due to voids in the tissue comes from (i) comparative analysis of thousands of diffraction patterns within each scan and (ii) correlated analysis of SAXS and WAXS data simultaneously. Indeed, in our earlier analysis of SAXS from tissues (Nepal *et al.*, 2022 ▸), the possibility of SAXS being due to voids was never considered. Nevertheless, when analyzed in the broader scattering context, the evidence, as we will outline below, is clear.

## 2. Methods

### 2.1. Sample preparation, data collection and processing

Tissue samples from Alzheimer’s disease cases were prepared at the Massachusetts Alzheimer’s Disease Research Center (MADRC) at the Massachusetts General Hospital with standard neuropathological processes (Liu *et al.*, 2016 ▸) to obtain sections 20 µm in thickness with chemical and physical properties typical of histological sections. These unstained sections are thicker than the usual histological sections in order to increase the volume of the material irradiated. Serial sections stained for the presence of Aβ or tau were used as guides to select regions for examination with X-ray microdiffraction. Tissue sections were spread on 12 µm-thick, 1 cm × 1 cm mica films and mounted on sample holders printed to LiX specifications (see below).

**Figure 1:**
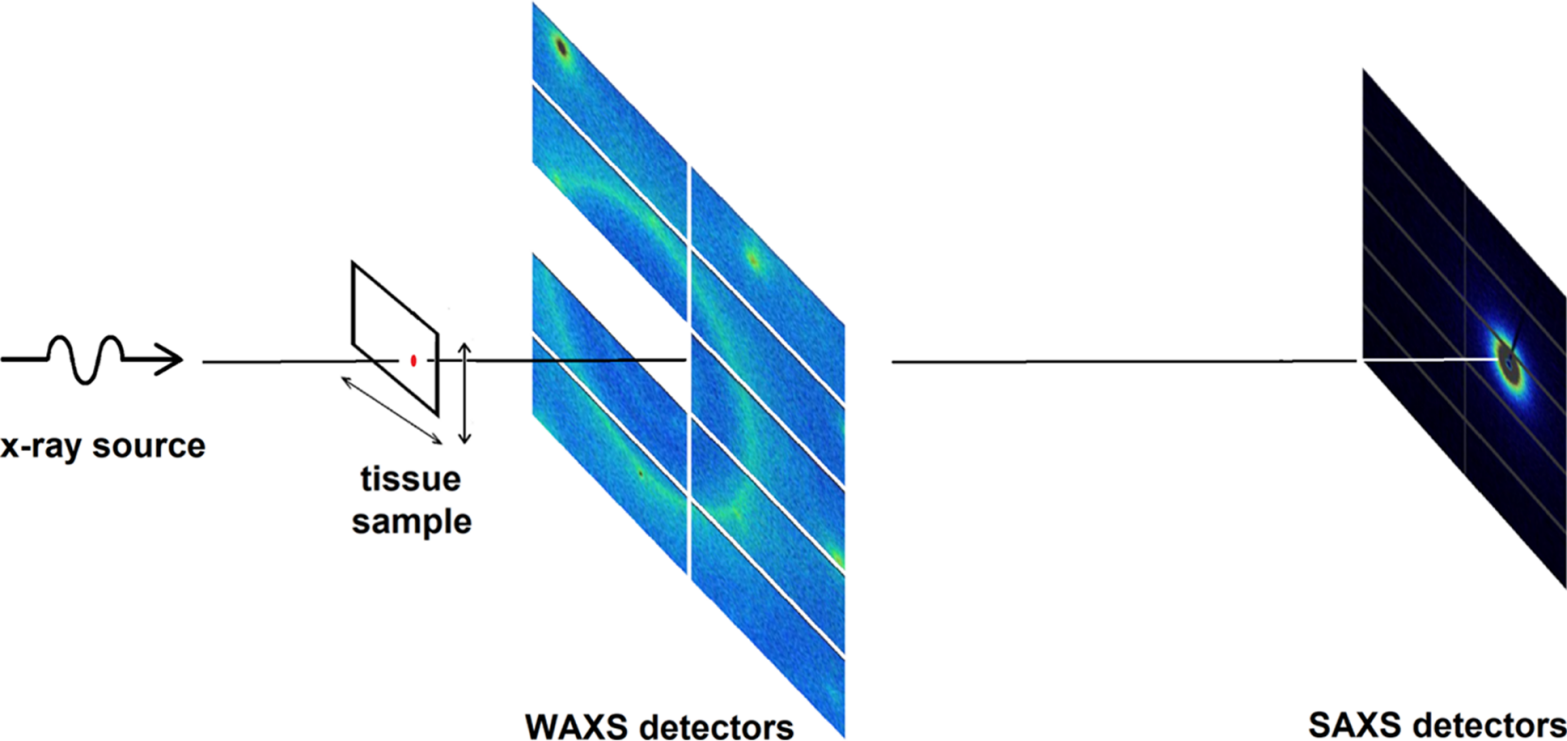
Scanning X-ray microdiffraction experiment. Several thousand diffraction patterns are collected from a specimen by stepping a suitable sample (*e.g.* tissue section) across an X-ray microbeam, collecting a complete diffraction pattern at every grid point on a square grid to build up an array of patterns that provide information on the distribution of constituents across the sample. Data across a wide range of scattering angles are collected simultaneously by utilizing WAXS and SAXS detectors in a single experiment.

Data collection was performed at the LiX beamline at the NSLS-II synchrotron source at Brookhaven National Laboratory (Yang *et al.*, 2020 ▸, 2022 ▸). The sample holders were mounted directly on the LiX beamline stage under vacuum. Tissue sections mounted on mica were scanned with a 5 µm-diameter X-ray microbeam to collect scattering patterns as a function of position on a square grid with 5 µm step size. Scans were performed over a rectangular area of between 300 × 300 µm^2^ (3600 diffraction patterns) and 600 × 600 µm^2^ (14 400 diffraction patterns). An exposure time of 0.5 s was used and (with data transfer and sample step) roughly 0.8 s was required per exposure, or about 48 min for a 300 × 300 µm^2^ region of interest (ROI). Data were collected on SAXS and WAXS detectors simultaneously (see Fig. 1 ▸), circularly averaged, and then merged using LiX-specific software. Required corrections including geometric corrections, transmission/scattering thickness corrections *etc.* were accounted for using extensively tested protocols from LiX (Yang *et al.*, 2020 ▸; Yang, 2013 ▸). Intensities were estimated at 570 *q* values over the range 0.005 < *q* < 2.7 Å^−1^.

Radiation damage was evaluated by multiple scans of the same tissue regions to ensure that scattering from fibrils was not changed by X-ray exposure. The comparison of data within a single scan of an ROI provides multiple opportunities for evaluation and correction of errors due to scaling in intensity. With regards to potential impact in the variation of sample thickness and dehydration, all correlations between WAXS intensity and the slope of equation (1) indicate minimal impact. On rare occasions, where the thickness varies across an ROI (for instance at the edge of tissue), the effect is immediately recognized. The diffraction patterns were preprocessed to eliminate scattering from mica (Bashit *et al.*, 2022 ▸).

## 3. Results

### 3.1. Form of observed scattering patterns

**Figure 2:**
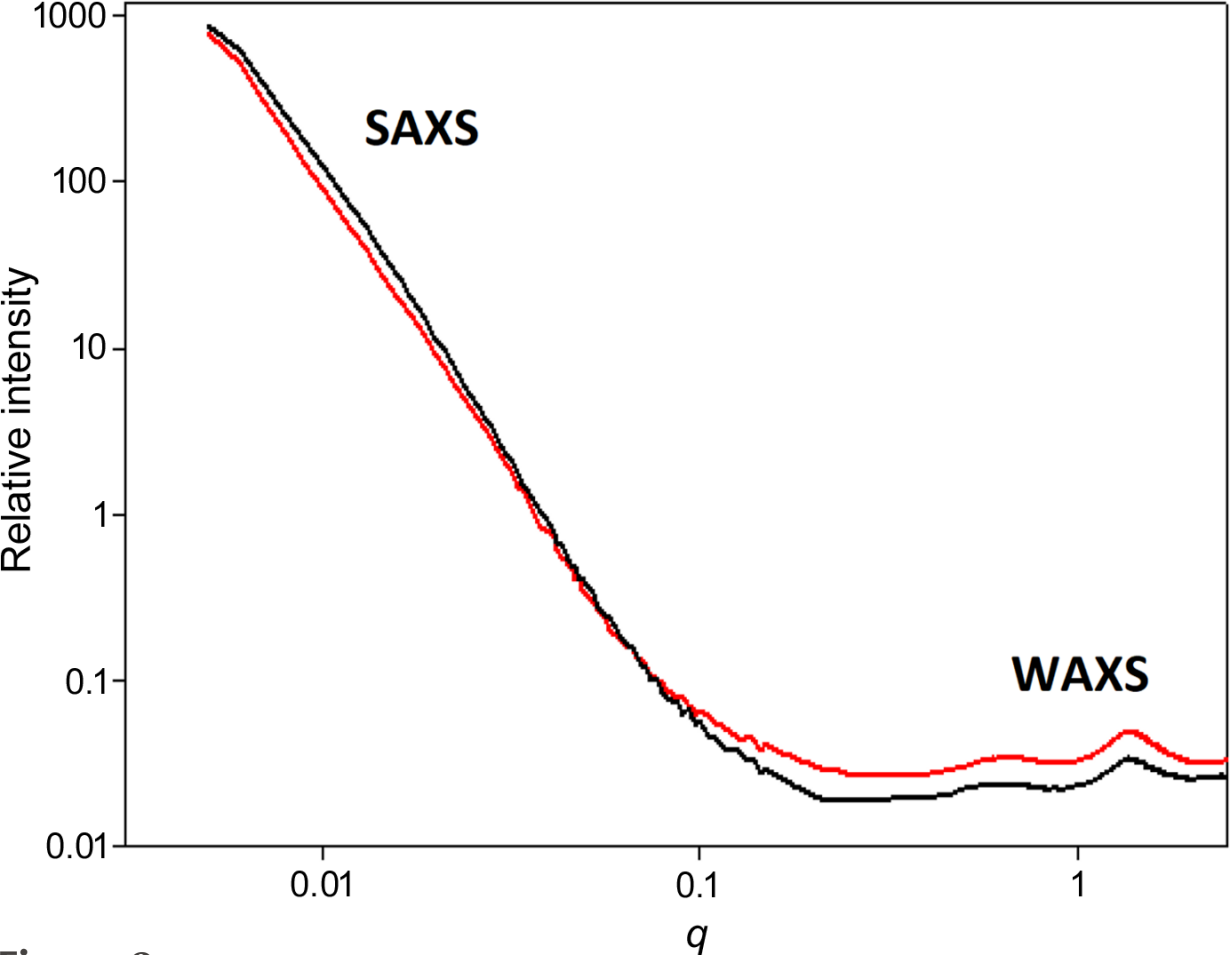
Circularly averaged intensity: log–log plots of intensity versus momentum transfer, *q*, for two patterns from a single scan of a thin section of human brain tissue. Intense small-angle scattering exhibits a linear, power-law behavior. In many cases, those patterns exhibiting the most intense wide-angle scattering exhibit weaker small-angle scattering.

Fig. 2 ▸ is a plot of the merged SAXS and WAXS intensities for two tissue locations from the same scanning ROI. The plots have several characteristics that are common to many of the patterns we have observed. First, the log of intensity is roughly linear with log(*q*) in the range 0.007 < *q* < 0.07 Å^−1^. The exponent *p* [equation (1)] was observed to fall between 3 and 4 in most cases. Second, the relative intensities of the patterns may invert between the SAXS and WAXS regions, with patterns more intense in WAXS being less intense in SAXS. This inversion was a surprise. A higher intensity in the broad WAXS peak indicates that the macromolecular structures are more densely packed at those locations, but the corresponding lower SAXS intensity suggests the opposite.

As shown below, the SAXS intensity is due to the presence of sub-micrometre-sized voids in the tissue. The linear relation in the log–log plot breaks down at very small *q* (<0.0075 Å^−1^) due to the limited size range of these voids. At large *q* (>0.07 Å^−1^) the linear dependence also breaks down, in this case due to contributions from other constituents of the scattering volume. At wider angles, the internal structure of macromolecular constituents dominates the observed scattering and gives rise to broad peaks at ∼10 and ∼4.7 Å spacings (*q* ≃ 0.6 and 1.34 Å^−1^, respectively). Guinier plots (not shown) of the patterns are non-linear.

### 3.2. Intensities in the SAXS and WAXS regimes are uncorrelated

**Figure 3:**
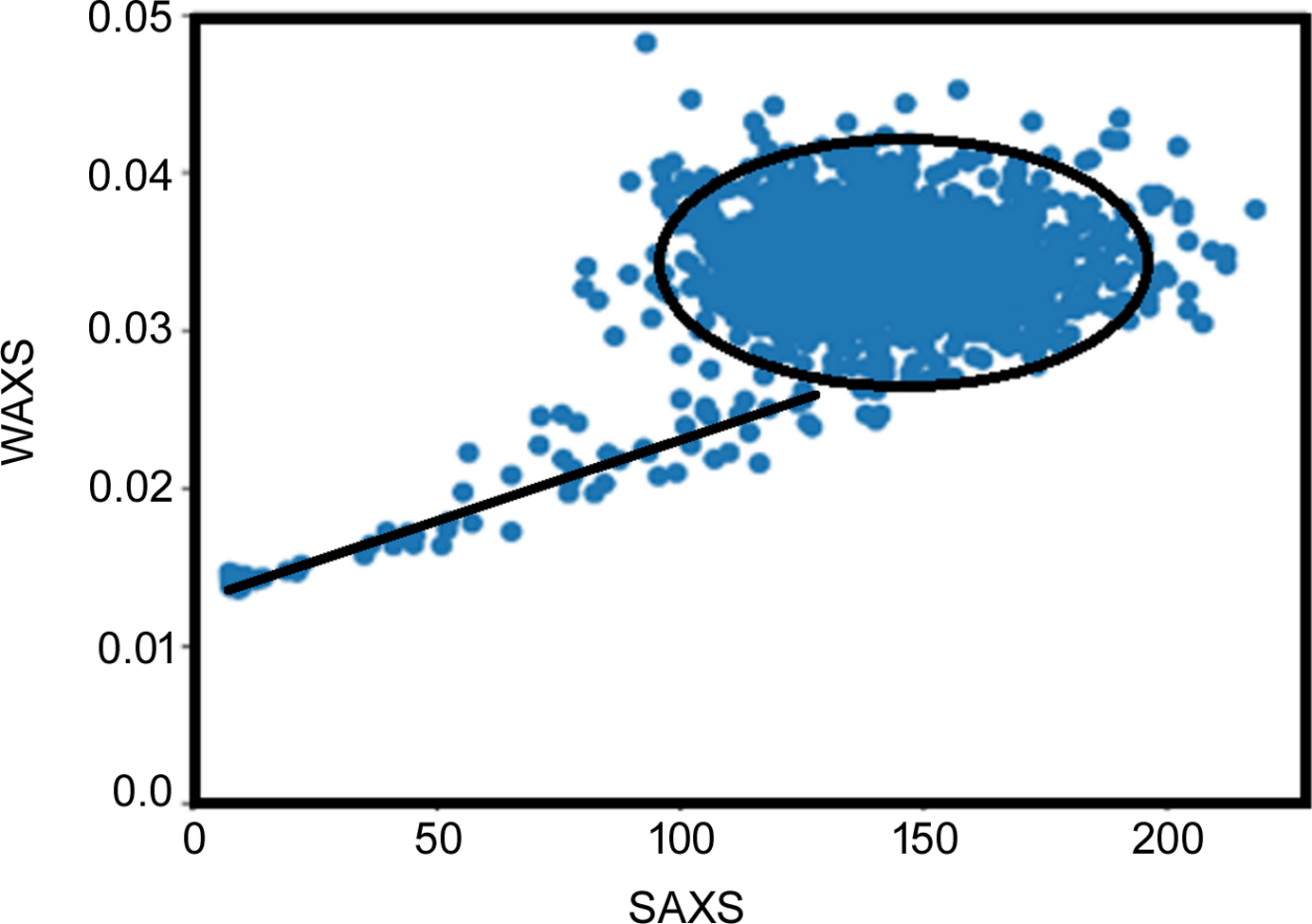
There is no correlation between the intensity of the SAXS and WAXS from tissue in a single ROI. Each point in this plot represents a pattern in the scan (3721 patterns). Scattering from tissue falls within a compact elliptical region with no apparent correlation between intensities in the SAXS and WAXS regimes. By contrast, the intensities of scattering from mica (outside the bounds of the tissue section) fall along a straight line, exhibiting the expected correlation between SAXS and WAXS intensities in the absence of tissue. Data are the intensities at *q* = 0.01 Å^−1^ (SAXS) and *q* = 1.34 Å^−1^ (WAXS).

**Figure 4:**
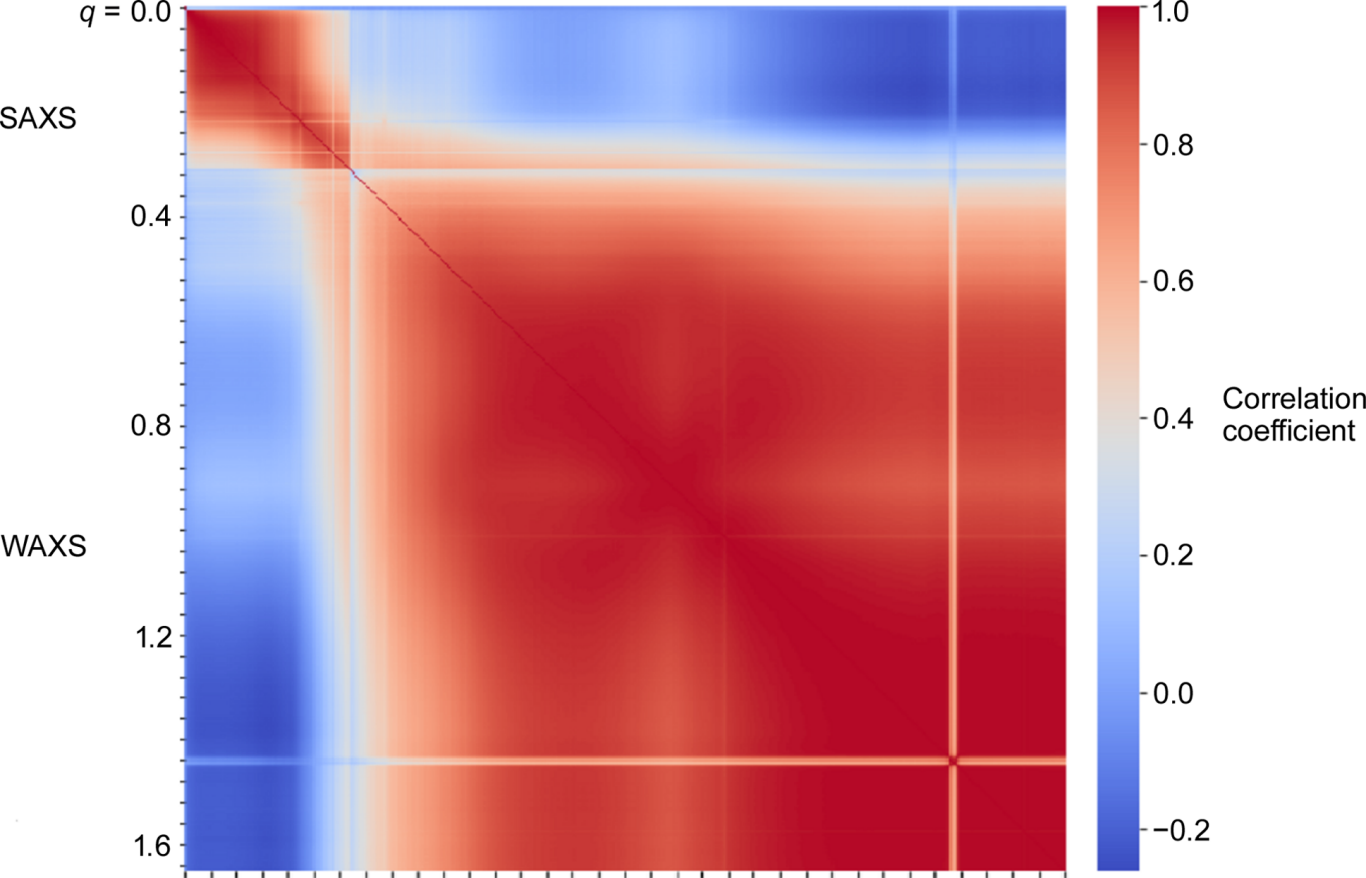
Heat map of a 570 × 570 matrix illustrating the correlation between intensities at 570 scattering angles. The relative intensities in the 3721 different scattering patterns at each *q* value are compared with the corresponding intensities at different *q* values. Across the SAXS regime the intensities in the 3721 patterns are well correlated (red square in the upper left of this heat map). Similarly, the intensities across the WAXS regime are well correlated with one another (large red region). But comparison of the distribution of intensities in the SAXS regime with that in the WAXS regime demonstrates that they are uncorrelated or anti-correlated (blue rectangles).

Comparison of scattering from different diffraction patterns from a single scan indicates that the intensity of scatter in the SAXS regime is poorly correlated with that in the WAXS regime (Table 1 ▸, Figs. 3 ▸ and 4 ▸). As a graphical demonstration of this, for each pair of scattering angles, (*q_i_*, *q_j_*), we used the distribution of relative intensities for all 3721 diffraction patterns in a 61 × 61 scan to calculate the correlation of intensity distribution in the scattering patterns for all 570 *q* values in each pattern. This correlation matrix is visualized as a heat map with 570 × 570 elements in Fig. 4 ▸. While the matrix demonstrates strong positive correlation (shown in red) of intensities within the SAXS regime (0.005 < *q* < 0.3 Å^−1^) as well as within the WAXS regime (0.3 < *q* < 2.7 Å^−1^), there is essentially no correlation of the intensity distributions between SAXS and WAXS (shown in blue in the heat map). This provides strong evidence that the intensity in the SAXS regime is due to structural features distinct from those giving rise to intensity in the WAXS regime.

### 3.3. Origin of the power-law behavior of the SAXS intensity

**Figure 5:**
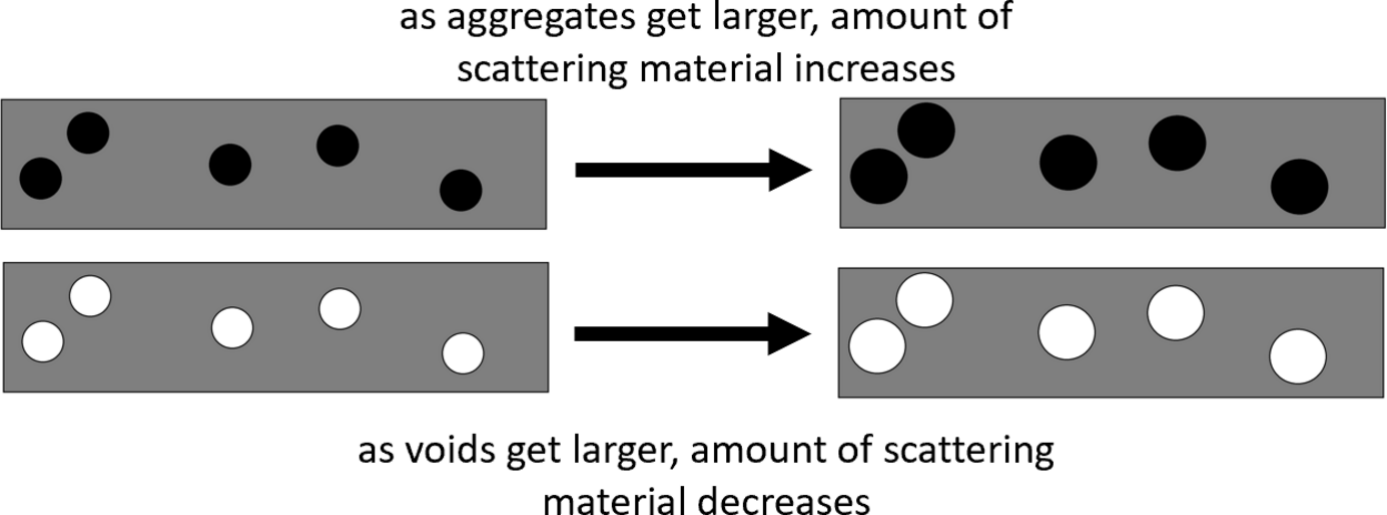
Diagram illustrating the impact of aggregates or voids distributed throughout tissue.

Babinet’s principle (*e.g.* Born & Wolf, 2013 ▸) states that scattering from a ‘hole’ (void) in a material is indistinguishable from scattering from a dense ‘inclusion’ of the same size, shape and contrast. While dense inclusions of the same size, shape and contrast as a void would generate indistinguishable data for any one diffraction pattern, when scattering from thousands of patterns is compared, a comparison of intensities in the SAXS and WAXS regimes can distinguish between these two possibilities. Small-angle scattering is due mainly to fluctuations in electron density on the nanometre length scale as might be generated by the presence of either aggregates or voids. As we will demonstrate below, an increase in the size of these scattering objects (aggregates or voids) would increase the (negative) slope of the SAXS log–log curve. The wide-angle scattering is due mainly to the partially ordered atomic arrangements of the macromolecular materials in the irradiated volume. As diagrammed in Fig. 5 ▸, if the tissue contains dense aggregates, an increase in the size of those aggregates would increase both the exponent, *p*, of the small-angle log–log curve and the intensity in the WAXS regimes. If the tissue contains voids, an increase in the size of those voids would also increase the exponent, *p*, of the SAXS curve but would decrease the scattering intensity in the WAXS regime. In most cases, we have observed the latter. We conclude that the behavior of the collective SAXS/WAXS curves in the ROI scans can best be explained by the presence of voids.

**Figure 6:**
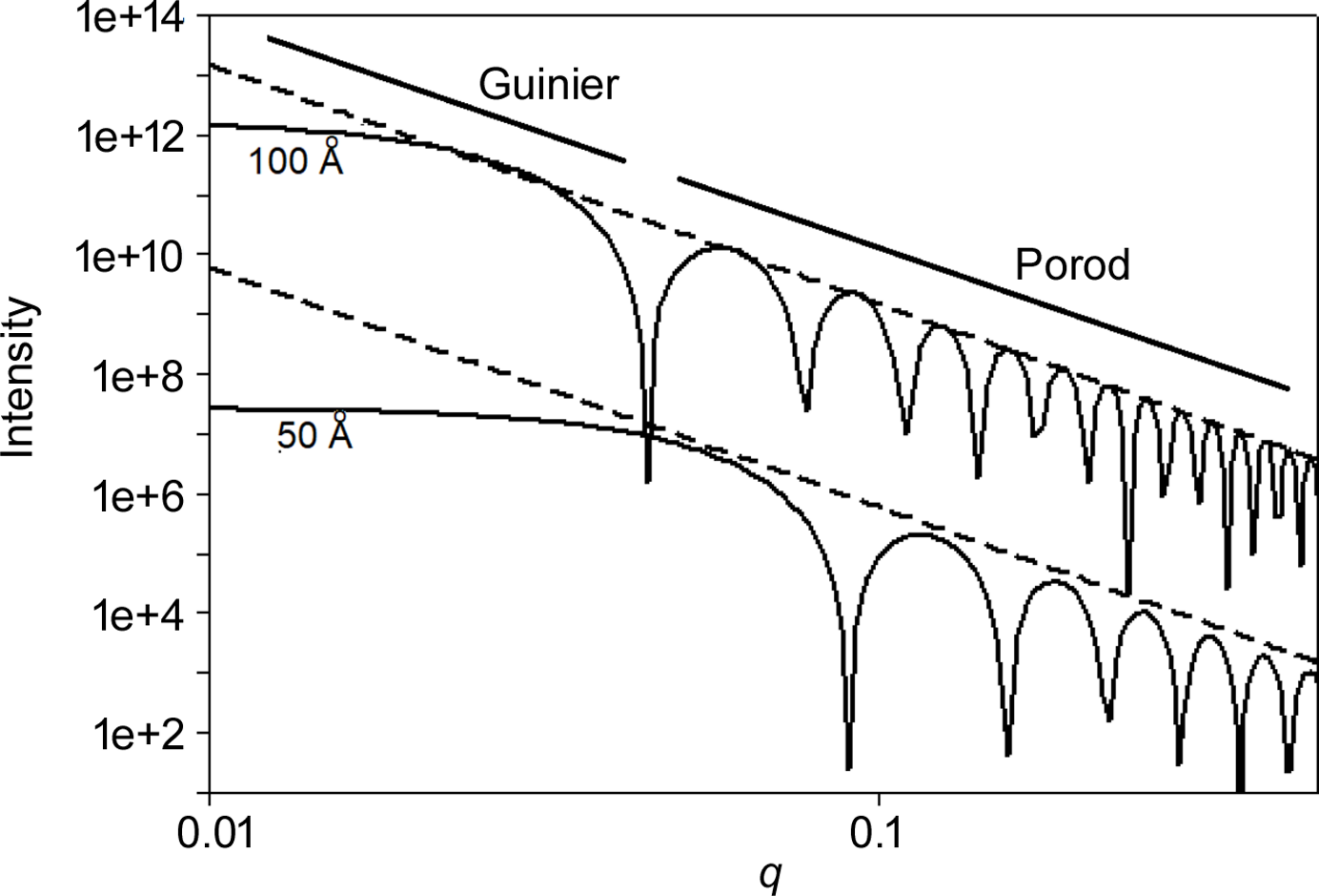
Log–log plot of *I*(*q*) for spheres of 50 and 100 Å radius calculated from the exact formula for scattering from a sphere (Guinier, 1963 ▸). A straight line through the peaks has a slope of −4 independent of the radius of the sphere.

Small-angle scattering, often used to obtain shape and size information about globular molecules in solution (Svergun & Stuhrmann, 1991 ▸; Svergun & Koch, 2003 ▸; Koch, Vachette & Svergun, 2003 ▸), can be divided into the Guinier regime at very low *q* and the Porod regime at somewhat larger *q* (Glatter & Kratky, 1982 ▸; Guilbaud & Saiani, 2011 ▸). This is illustrated in Fig. 6 ▸, which shows the predicted scattering intensity from a solid sphere as a function of *q*. This calculation is equally valid for a dense sphere or a spherical void surrounded by dense material. In the Guinier regime, for a monodisperse solution of particles, the log of intensity is linear as a function of *q*^2^ (Guinier plot) and the slope is frequently used to determine the radius of gyration of the scattering particles (Putnam *et al.*, 2007 ▸). As *q* increases into the Porod regime, the log of the intensity of periodic peaks in the scattering from a sphere decreases with log(*q*) linearly with a slope of −4 *independent of the radius of the sphere*. That is, the peak intensities vary with *q* as
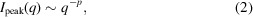
with the scattering exponent, *p*, equal to 4. Not only is this slope independent of the radius of the sphere, but it is also invariant for essentially all compact shapes (*e.g.* a short solid cylinder or a cube or another less regular shape). While intensity peaks from a compact object decay as *q*^−4^ through the Porod regime, intensity peaks from a very thin disc decay as *q*^−2^ and from a very thin rod as *q*^−1^. These observations are the basis of relating the slope of the log–log plot to the ‘dimension’ of the scattering object. Nevertheless, in scattering from many heterogeneous materials, the subsidiary peaks are not observed, and the scattering intensity exhibits a power-law behavior with a slope that may be integer or non-integer in value (Roe, 2000 ▸).

Fractals were introduced into polymer and colloid science to explain non-integer power-law behavior of SAXS scattering (*e.g.* Ogawa *et al.*, 1989 ▸; Suzuki *et al.*, 1997 ▸; Martin & Ackerson 1985 ▸; Martin & Hurd, 1987 ▸; Anitas, 2019 ▸) which follows directly from the scale-free distribution of lengths intrinsic to fractal geometry. Here we will show that our observations of non-integer power-law SAXS intensity can be explained by a scale-free size distribution of a heterogeneous ensemble of voids. Since all compact objects are predicted to generate intensity that decays as *q*^−4^, it seems counter-intuitive that ensembles of different sized objects might generate scattering that exhibits a power-law behavior with slope not equal to −4. However, as we will show, non-integer slopes may arise in scattering from a heterogeneous population of scattering objects because the transition from Guinier regime to Porod regime occurs at different *q* values for different sized objects.

**Figure 7:**
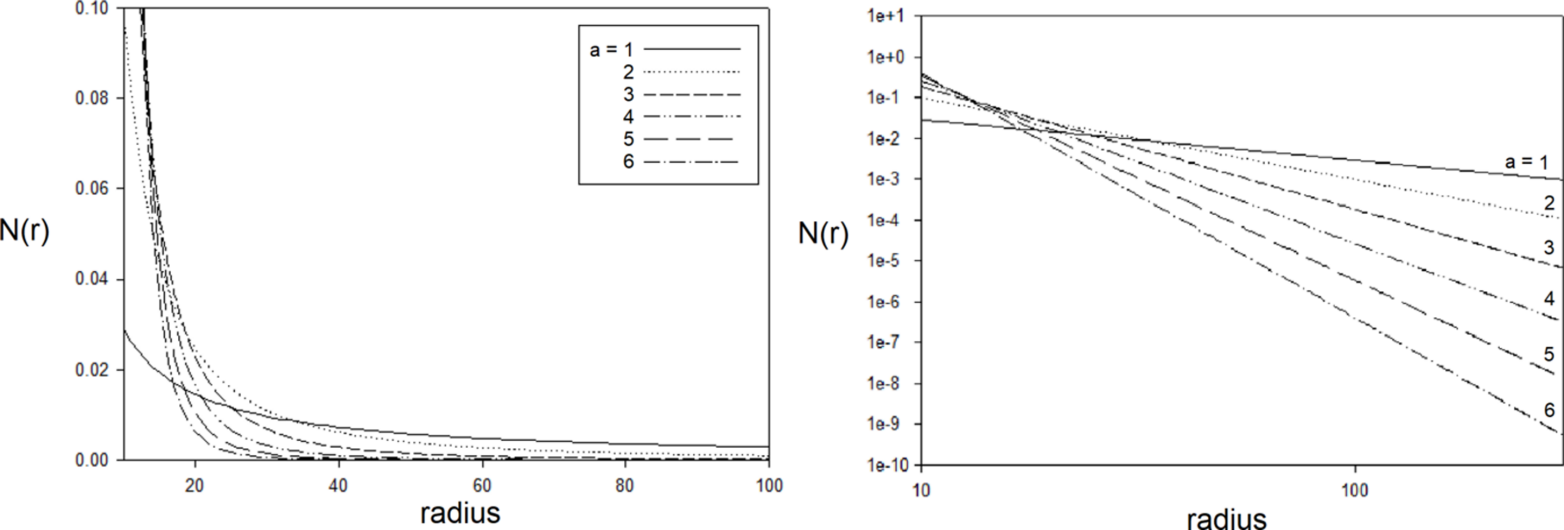
Number density of spheres, *N*(*r*) = *r*^−*a*^, as a function of radius normalized so that each distribution has the same total number of spheres over the radius range 10–300 Å. When the radial exponent *a* increases, the number of large spheres decreases, as does the average radius of spheres within the ensemble. Left: linear plot. Right: log–log plot exhibiting the power-law property of the distribution chosen.

**Figure 8:**
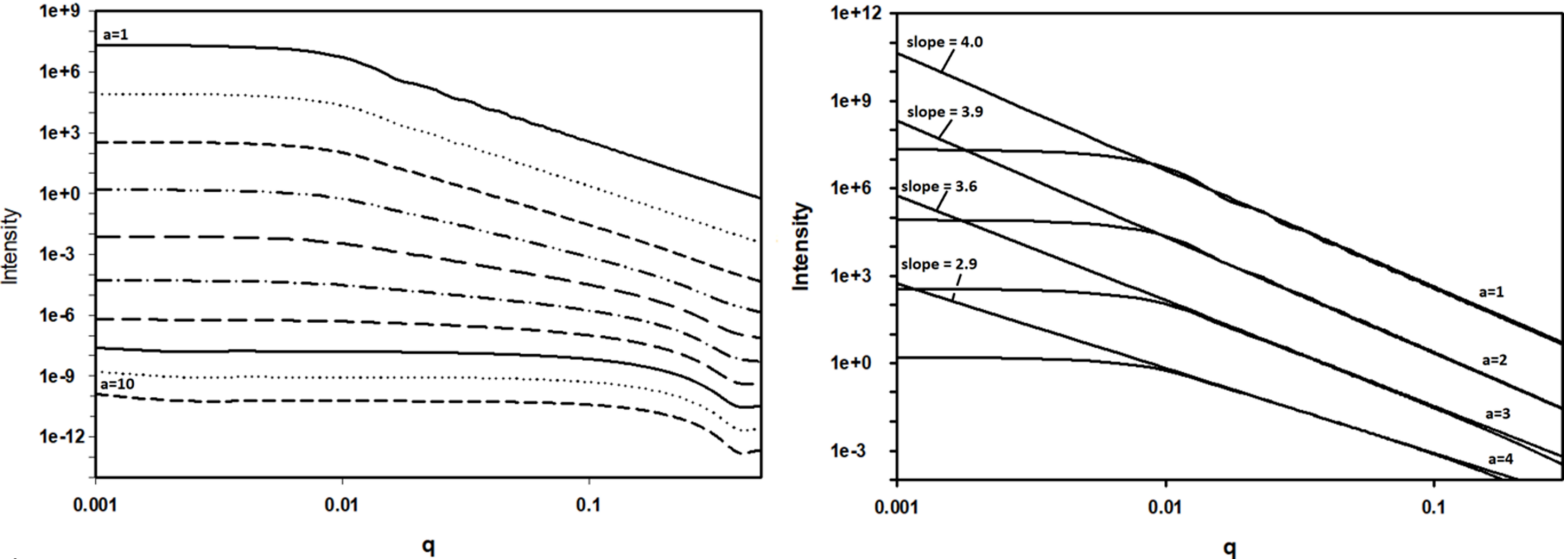
Calculated scattering from ensembles of spheres with number density varying as a function of radius, *N*(*r*) = *r*^−*a*^. The heterogeneous distribution of radii fills in the troughs in the calculated scattering from a single sphere (seen in Fig. 6 ▸). It also mixes scattering patterns that have different *q*_min_ below which the power-law behavior ends. That leads to scattering with different apparent slopes. Left: scattering from ten ensembles with *a* = [1,10]. Right: linear fits to the calculated intensities for *a* = [1,4].

**Figure 9:**
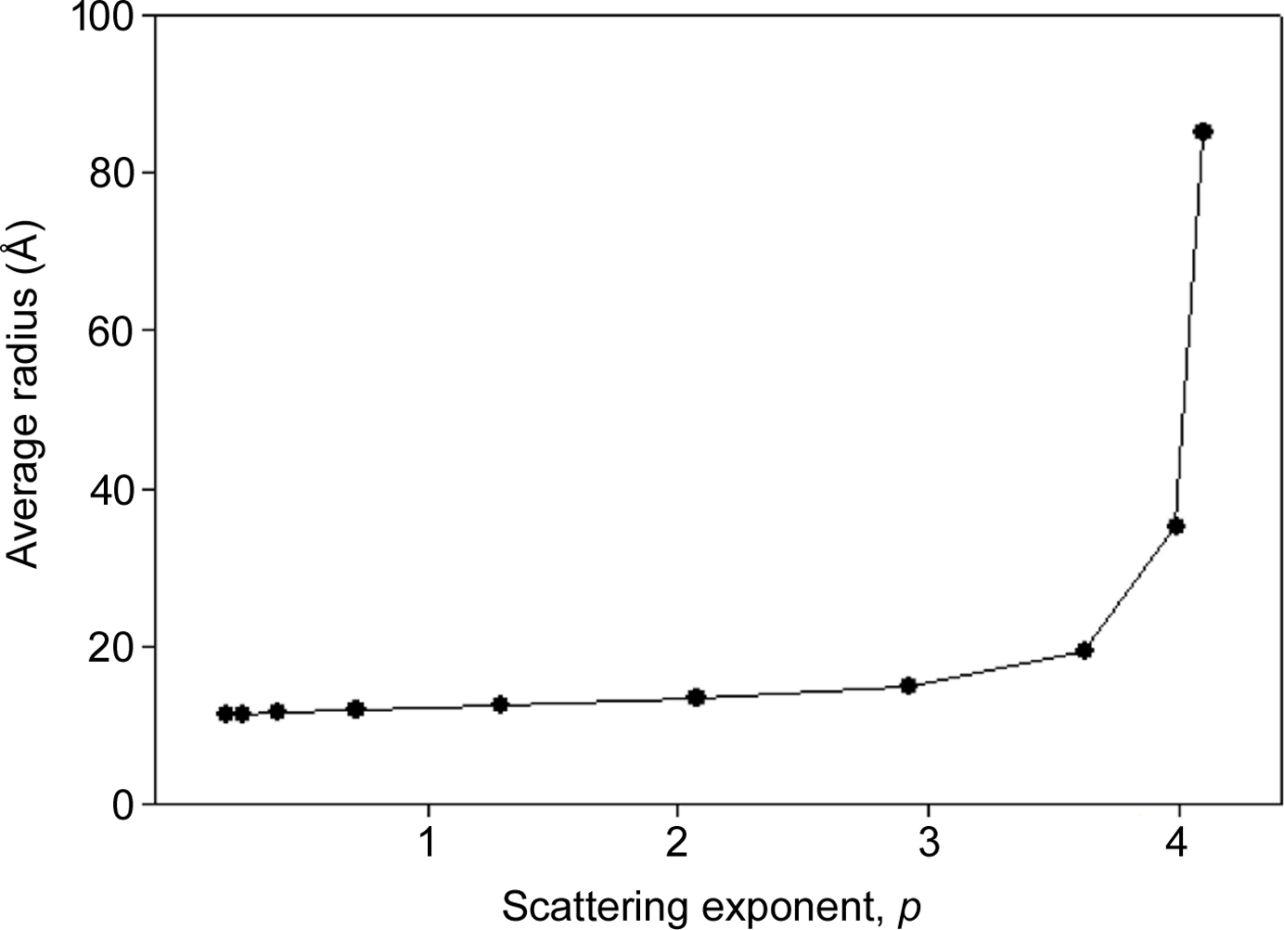
Average radius as a function of scattering exponent in an ensemble of spherical voids with a scale-free distribution of sizes and *r*_min_ and *r*_max_ values equal to 10 and 300 Å respectively. As the scattering exponent increases, the (negative) slope of the log(*I*) versus log(*q*) curve increases, indicating an increase in the average size of scattering objects.

Consider an ensemble of voids where their sizes are distributed in a scale-free manner. Scale-free here simply means that the distribution of scattering object size exhibits a power-law behavior, *i.e.* the log of the abundance of scattering objects decreases linearly with the log of scattering object size. In other words, their number density varies as a function of radius as
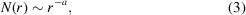
where we will refer to ‘*a*’ as the ‘radial exponent’. This distribution can only extend over a limited range of radii since *r* → 0, *N* → ∞. To demonstrate the scattering properties of scale-free ensembles, we chose to calculate the scattering from an ensemble of spheres with radial exponents *a* ∈ [1,10] and *r*_min_ and *r*_max_ values of 10 and 300 Å, respectively. These distributions were normalized so they each had the same total number of scattering objects as plotted in Fig. 7 ▸. The scattering predicted to arise from these ten distributions was calculated using the exact formula for scattering from a sphere (Guinier, 1963 ▸) and is shown in Fig. 8 ▸. The distribution of radii chosen for these calculations results in predicted scattering that approximates a linear log–log dependence with a non-integer slope over a limited range of *q*. As the radial exponent *a* increases, large spheres become less common, the average radius of spheres in the ensemble decreases and the slope of the scattering curve becomes more shallow (less negative) as shown in Fig. 8 ▸. As seen in Fig. 9 ▸, the average radius of scattering particles (in this case voids) increases as the log–log curve becomes steeper (greater negative slope), as one would expect from the intrinsically ‘reciprocal’ nature of scattering space.

**Figure 10:**
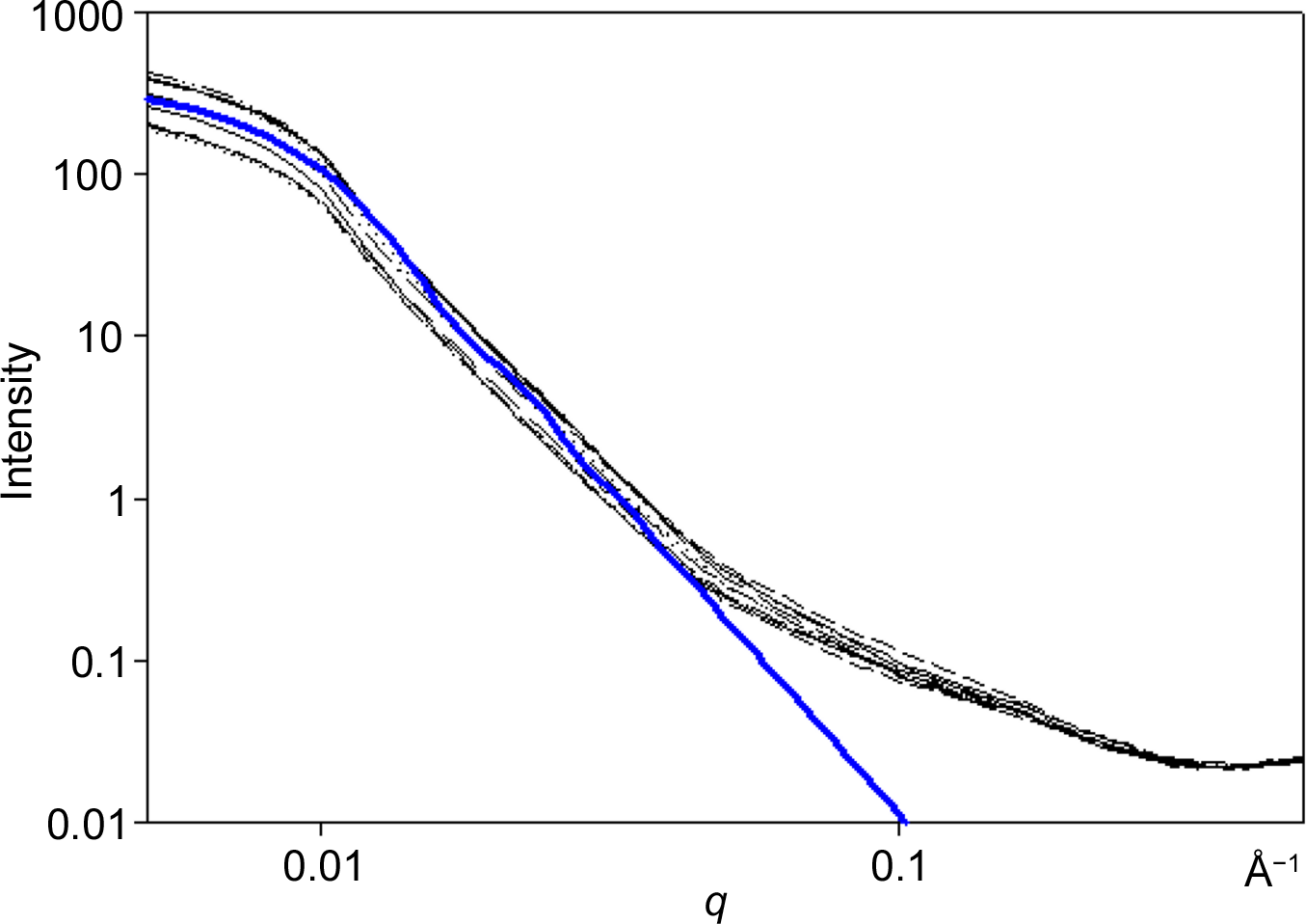
Comparison of calculated versus observed intensity. The black curves are scattering from a tau lesion in a thin section of human brain tissue. The blue curve is that calculated for scattering from an ensemble of spheres with number density *N*(*r*) ∼ *r*^−*a*^ with *a* = 3.2, *r*_min_ = 100 Å and *r*_max_ = 500 Å. The leveling off of intensity at low *q* (<0.01 Å^−1^) indicates that there are few voids with radii greater than ∼500 Å in the scattering volume.

The power-law behavior of the intensity extends to a minimum *q* value, *q*_min_, below which it plateaus. This *q*_min_ decreases as the size of the largest scattering particles in the ensemble increases. Minimizing the difference between calculated and observed provides an estimate of *r*_max_ for a given sample. As an example, Fig. 10 ▸ shows the intensity calculated for *r*_min_ = 100 Å, *r*_max_ = 500 Å and radial exponent *a* = 3.2 compared with the SAXS intensities observed from a scan of a tau-containing tissue section. A leveling off of the log–log plot at small *q* values (as is apparent in Fig. 10 ▸) is observed in most patterns collected in this study and suggests that the voids in these fixed tissues are seldom – if ever – greater than ∼0.1–0.2 µm in diameter. However, the presence of a distinct population of much larger voids that would scatter at unobservably small angles cannot be ruled out by our data.

In summary, the non-integer power-law behavior of *I*(*q*) at small scattering angles is consistent with that expected for a heterogeneous population of compact objects (objects with relatively low axial ratios) with scale-free size distribution. The average size of scattering objects that generate this intensity can be estimated from the slope of the log–log plot. As the average size of scattering objects increases, the negative slope of the log–log plot will increase (Fig. 10 ▸).

### 3.4. Variation of SAXS and WAXS intensity with the slope of the log(*I*) versus log(*q*) curve

**Figure 11:**
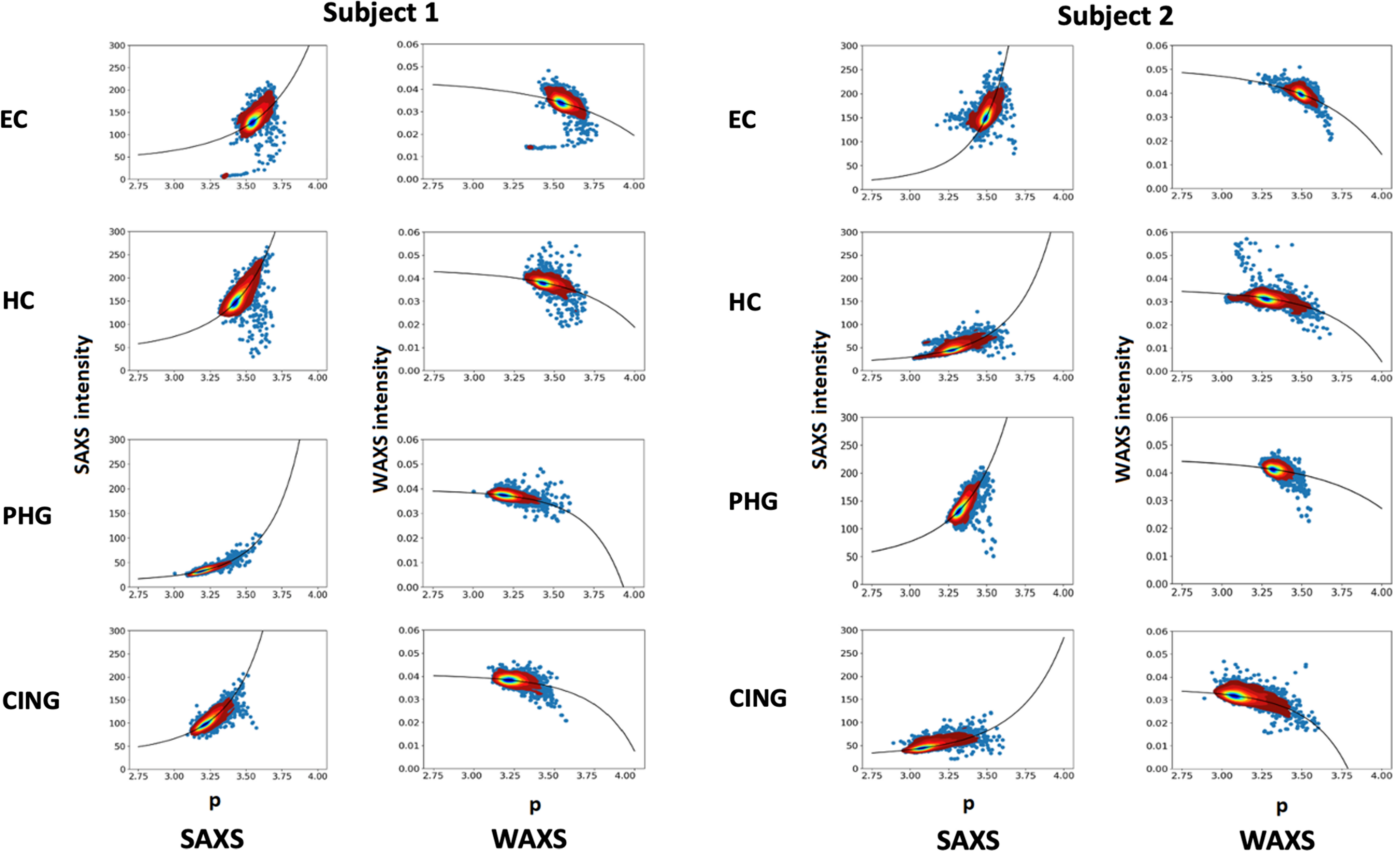
Scatter plots illustrating the correlation between the scattering exponent, *p*, and the SAXS and WAXS intensities for the ROIs in the two advanced Alzheimer’s cases (Braak VI, Thal 5) detailed in Table 2 ▸. Each scatter plot represents 3721 diffraction patterns. The regions of greatest density of data points are contoured to provide a better visualization of the distribution of points; the individual points observed outside the contours are outliers, often representing patterns from lesions or regions of the scan that fell outside the extent of the tissue (*i.e.* mica background). The solid black lines are best fits to the data for equations (4) (SAXS data) and (5) (WAXS data) using parameters given in Table 2 ▸. EC – entorhinal cortex; HC – hippocampus; PHG – posterior parahippocampal gyrus; CING – anterior cingulate cortex.

As shown in Table 1 ▸, there is a positive correlation between the SAXS intensity and the scattering exponent, *p*, of the log–log plots and a negative correlation between WAXS intensity and *p*. Scatter plots demonstrating this for eight ROI scans are shown in Fig. 11 ▸. Fig. 9 ▸ relates the scattering exponent to the average radius of scattering objects, which makes it possible to predict changes in the average radius of voids that give rise to the observed scattering. Scatter plots of SAXS and WAXS intensities versus the power-law exponent (Fig. 11 ▸) can be used to test those predictions and thereby generate information about the basis of changes in the SAXS patterns across the ROIs that have been scanned. Since scattering from all compact objects (low axial ratio objects) is roughly the same in the Porod regime, we will approximate scattering from the irregular voids in dehydrated tissue as that due to equivalently sized spheres.

As shown in Appendix *A*[App appa], if we assume that the size and number of voids present in a scattering volume vary across an ROI, the intensity in the SAXS regime should vary as
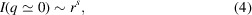
where the SAXS exponent, *s*, would equal 6 if the *number* density of voids is constant and 3 if the *volume* density of voids is constant. As seen in Table 2 ▸ and Fig. 11 ▸, fitting the data in individual ROIs results in estimates of this exponent between 2 and 6, suggesting that as the average radius of a void increases the number density of voids will typically decrease. This might be expected if voids tend to coalesce during the dehydration process.

The intensity of scattering in the WAXS regime will depend on the volume occupied by macromolecular material. That intensity should *decrease* as the radius or number of voids increases as diagrammed in Fig. 5 ▸. As shown in the appendix, this can be modeled as
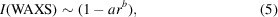
where ‘*ar^b^*’ is the volume fraction of voids in the scattering volume [equation (11) in Appendix *A*[App appa]] and ‘*b*’ is referred to as the WAXS exponent (Table 2 ▸). The terms *a* and *b* can be derived from fitting the data for all patterns in an ROI to equation (5) as in Fig. 11 ▸. The data in Fig. 11 ▸ are consistent with the functional forms of equations (4) and (5) but exhibit a magnitude of scatter that precludes accurate estimates of number density and volume fraction, and the estimates in Table 2 ▸ should be considered rough approximations.

### 3.5. Mapping variation of SAXS and WAXS intensity and slope of the log(*I*) versus log(*q*) curve

**Figure 12:**
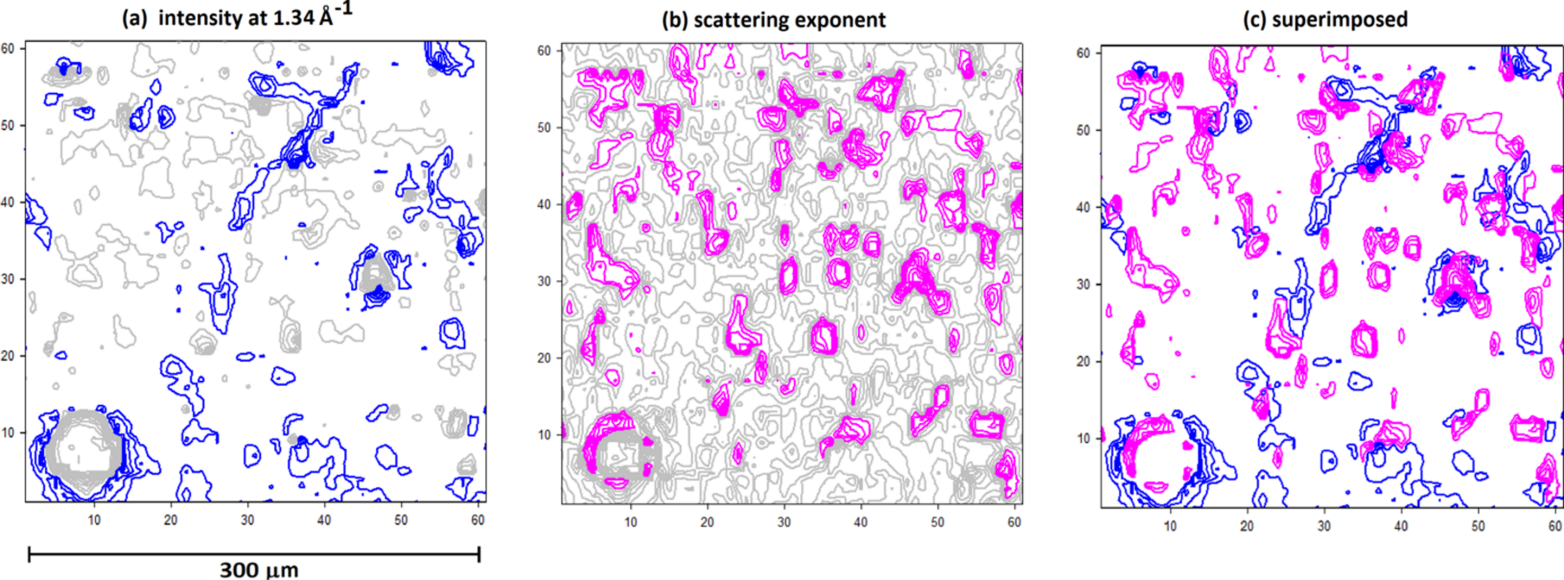
Contour plot of a 300 × 300 µm^2^ ROI from the entorhinal cortex of Sample 1 documented in the tables. (*a*) Blue contours represent the regions of high macromolecular density. (*b*) Magenta contours represent regions of highest scattering exponent (highest porosity). (*c*) Superposition of (*a*) and (*b*). The circular feature in the lower left is a cross section of a small blood vessel that intersected the section.

A key advantage of scanning microdiffraction is that any attribute of the scattering data can be mapped across an ROI. Mapping the intensity of wide-angle scattering as a function of position across the ROIs provides a visualization of the regions of greatest macromolecular density, which might include dense cellular debris, vascular walls, and Aβ and tau lesions. Fig. 12 ▸ is a contour map showing (in blue) the distribution of scattered intensity at 4.7 Å spacing (*q* = 1.34 Å^−1^) across a 300 × 300 µm^2^ ROI collected from the entorhinal cortex of an advanced Alzheimer’s case (Braak VI, Thal 5). Superimposed in magenta is the distribution of the scattering exponent, *p*, from the same scattering patterns. As shown in Fig. 9 ▸, the larger the scattering exponent, the larger the average size of the voids. Not surprisingly, regions containing the largest voids do not overlap the regions exhibiting the highest macromolecular density.

## 4. Discussion

The principal result reported here is that fixed, dehydrated human brain tissue is suffused with sub-micrometre-sized voids. This conclusion is based on the observation that, within the thousands of scattering patterns that make up the scan of an ROI, the small-angle log(*I*) versus log(*q*) plot is approximately linear with a (negative) slope that is correlated with the SAXS intensity and inversely correlated with the WAXS intensity. We have observed this behavior in dozens of ROIs in tissue from different parts of the brain and different subjects.

Significantly, this result could not have been reached from analysis of individual scattering patterns. Rather, it demonstrates the power of deciphering the physical properties of heterogeneous materials through the use of scanning microdiffraction. Furthermore, it required the coordinated analysis of both small-angle and wide-angle scattering. Observing that the intensities in the SAXS and WAXS regimes were not correlated to one another led to the realization that they derived from independent features of the scattering material. This was a key insight that made the interpretation of the patterns possible.

Additional information about the structure of the tissue was generated by modeling these voids as spheres for the purpose of calculating their scattering properties. The universal Porod-law behavior of compact objects (with axial ratios near unity) supports the notion that a heterogeneous ensemble of irregular shaped voids will scatter in ways similar to that of a heterogeneous ensemble of spheres as long as their average axial ratio is not too large. The success of a simple model for estimating SAXS and WAXS intensity as a function of the scattering exponent [equations (4) and (5), and Fig. 11 ▸] further supports this approach. The estimates of number density and volume fraction based on this model (Table 2 ▸) are approximate, but their relative values should reflect material properties.

While it is now clear that the SAXS intensities observed in scattering from fixed human brain tissue are dominated by the contribution of voids created during dehydration, the macromolecular constituents of the tissue also contribute observable intensity in the small-angle regime. Scattering from dense plaques containing a population of fibrils often includes weak peaks or shoulders reflective of the cross-sectional shape of the fibrils and the spatial correlations between proximal fibrils (Nepal *et al.*, 2022 ▸; Liu *et al.*, 2024 ▸). Our earlier analysis of SAXS from human brain tissues (Nepal *et al.*, 2022 ▸) did not recognize that a large fraction of the SAXS intensity was due to voids within the tissue. Whereas the correlation functions we derived from the SAXS data in that study were interpreted as due to cross-correlation between the embedded fibrils and the surrounding fibrils, it is now clear that the small-angle scattering includes a significant contribution from voids. The analysis presented here makes the separation of scattering due to voids from that due to fibrils and tissues possible. Moreover, as indicated by our data, these voids are largest and most prevalent in tissue where the density of cellular materials is lowest.

The linearity of the log–log *I*(*q*) curve over a substantial proportion of the small-angle regime in scattering from fixed human brain tissue appears similar to the power-law behavior widely attributed to fractals. It is observed in scattering from many other types of tissue and materials. A homogeneous population of scattering particles will not generate scattering of this form. The literature is rife with studies that show that power-law SAXS intensity distributions can be explained by a fractal-like structural material. But the material attribute giving rise to the power-law behavior is the scale-free nature of the size distribution of scattering objects. This size distribution was the basis for constructing the heterogeneous ensemble of voids used to model our data. The consequent power-law distribution of void sizes was shown to be consistent with our observations.

The existence of a power-law distribution of void sizes is not readily explained in terms of the processes that lead to the formation of voids during tissue dehydration. Nevertheless, the power-law nature of *I*(*q*) in the SAXS regime is sufficiently universal that one is tempted to conclude that a simple model may be sufficient to explain a wide range of phenomena. If, for instance, the probability of a void increasing in size becomes larger as the void increases in size, then a power-law distribution of void sizes may follow (Bak, 1996 ▸).

The capability of estimating the volume fraction of voids from scattering data provides a novel probe of the structure – and potentially structural degradation – of human brain tissue in disease. The distribution of voids across thin sections of human brain tissue undergoing neurodegradation is highly heterogeneous, with regions of plentiful voids interspersed with regions of few voids. The regions of high void density may act as biomarkers to identify regions that might have been subjected to significant neurodegeneration. When mapped over multiple ROIs spanning multiple brain regions, patterns in the distribution of these attributes could be correlated with the disease stage and may provide insights into the molecular processes of disease progression.

## Figures and Tables

**Table 1:** Correlation coefficients between the intensity at small angles (SAXS; *q* = 0.01 Å^−1^), the intensity at wide angles (WAXS; *q* = 1.34 Å^−1^) and the scattering exponent *p* [equation (1)] as observed in eight ROIs from Alzheimer’s subjects with advanced disease EC – entorhinal cortex; HC – hippocampus; PHG – posterior parahippocampal gyrus; CING – anterior cingulate cortex.

	Correlation coefficient		
Sample	SAXS versus *p*	WAXS versus *p*	SAXS versus WAXS
Subject 1 EC	0.556	−0.248	0.506†
Subject 1 HC	0.553	−0.447	0.190
Subject 1 PHG	0.884	−0.428	−0.170
Subject 1 CING	0.820	−0.409	−0.038
Subject 2 EC	0.466	−0.608	0.140
Subject 2 HC	0.663	−0.448	0.148
Subject 2 PHG	0.672	−0.436	0.051
Subject 2 CING	0.666	−0.548	0.129

† The positive correlation between SAXS and WAXS observed in Subject 1 EC is due to the presence of a portion of the ROI having no tissue, giving rise to outliers in the scatter plot that do not reflect the properties of the tissue (see Fig. 3 ▸).

**Table 2:** SAXS and WAXS exponents [equations (4) and (5)], average radius of voids, void number density, and void volume fraction as estimated from the relationship between the scattering exponent and the average radius of an ensemble (Fig. 9 ▸) and WAXS intensities [equation (11)] in eight ROIs from Alzheimer’s subjects with advanced disease The median radius was estimated from the slope of the log–log plot with linear fits of observed intensities as demonstrated in Fig. 8 ▸ (left) using an empirical formula derived from fitting the curve in Fig. 9 ▸: *r* = 12 + 26 exp(2*p* − 8.2), where *p* is the observed scattering exponent. EC – entorhinal cortex; HC – hippocampus; PHG – posterior parahippocampal gyrus; CING – anterior cingulate cortex.

Sample	SAXS exponent	WAXS exponent	Median radius (Å)	Number density (Å^−3^)	Volume fraction
Subject 1 EC	2.1	1.0	20.6 Å	11.0 × 10^−6^	0.41
Subject 1 HC	3.0	1.8	18.5	8.0 × 10^−6^	0.21
Subject 1 PHG	3.9	3.8	16.2	4.9 × 10^−6^	0.09
Subject 1 CING	3.9	2.9	16.4	5.6 × 10^−6^	0.10
Subject 2 EC	5.5	1.5	19.8	10.7 × 10^−6^	0.35
Subject 2 HC	3.3	2.3	16.9	9.3 × 10^−6^	0.18
Subject 2 PHG	3.4	1.3	17.5	9.2 × 10^−6^	0.21
Subject 2 CING	2.4	3.5	15.4	10.5 × 10^−6^	0.16

## Appendix A

The small-angle (*q* ≃ 0) scattered intensity from an ensemble of *N* spheres is proportional to the sixth power of their radius [*I*(*q* ≃ 0) ≃ *Nr*^6^ (Guinier, 1963 ▸)]. If two scattering patterns are collected from regions where the number density of spheres is the same but the average radius, *r*, of the spheres is different, then the ratio of the intensities of small-angle scattering in the two scattering volumes should be proportional to *r*^6^. If the number of spheres changes as the average radius increases, then the increase in the intensity of small-angle scattering might be proportional to a different power of the radius. For instance, if the average density of the scattering volume remains the same while the average radius of the scattering objects increases, then the number of scattering objects would necessarily decrease by an amount proportional to *r*^−3^. In that case, the increase in the intensity of small-angle scattering would be proportional to *r*^3^ (rather than *r*^6^). In other words, as *r* changes, the SAXS intensity will change as

when the number density of voids is constant and

when the density of tissue is constant (number of voids decreases as *r* increases). In the general case,

where the SAXS exponent, *s*, can be estimated from the relationship between SAXS intensity and average void size. As seen in Table 2 ▸ and Fig. 11 ▸, fitting the data in individual ROIs results in estimates of the exponent between 2 and 6, suggesting that as the average radius of a void increases the number density of voids decreases. This might be expected if voids tend to coalesce during the dehydration process.

The intensity of scattering in the WAXS regime will depend on the volume occupied by macromolecular material. That should decrease as the radius or number of the voids increases as diagrammed in Fig. 5 ▸. In other words, the wide-angle intensity will be roughly proportional to the scattering volume, *V*_s_, minus the volume of voids, *V*_v_:

Assuming the total volume of voids can be approximated as the volume of the average sized void times the number of voids, *N*, and approximating the volume of a void as that of a sphere of equivalent size:

We can re-write this in terms of the number density of voids, *n* = *N*/*V*_s_,

Data from the scattering patterns in a single ROI are consistent with a curve of the form

where ‘*ar^b^*’ is the volume fraction of voids in the scattering volume [equation (11)] and ‘*b*’ is referred to as the WAXS exponent (Table 2 ▸). The terms *a* and *b* can be derived from fitting of data for all patterns in an ROI as in Fig. 11 ▸. Although the data exhibit a magnitude of scatter that makes it impossible to precisely define the functional form of the relationship between WAXS intensity and radii of voids, the observations are consistent with equation (10) and make an estimation of the number density of the voids possible. Combining equations (9) and (10), we have

This relation was used to estimate the volume fraction of voids in the tissue and that average for each ROI examined is shown in Table 2 ▸. Predictions for eight ROIs are plotted as solid lines in the scatter plots in Fig. 11 ▸. Similar results were obtained for dozens of other ROIs and will be reported in subsequent publications.

## References

[bb1] Anitas, E. M. (2019). *Small-Angle Scattering (Neutrons, X-rays, Light) from Complex Systems: Fractal, Multifractal Models for Interpretation of Experimental Data.* Springer.

[bb2] Avnir, D. D., Farin, D. & Pfeifer, P. (1984). *Nature*, **308**, 261–263.

[bb3] Bak, P. (1996). *How Nature Works*. Springer Verlag.

[bb4] Bashit, A. A., Nepal, P., Connors, T., Oakley, D. H., Hyman, B. T., Yang, L. & Makowski, L. (2022). *Front. Neurosci.* **16**, 909542. 10.3389/fnins.2022.909542 PMC9198601 35720706

[bb5] Born, M. & Wolf, E. (2013). *Principles of Optics: Electromagnetic Theory of Propagation, Interference and Diffraction of Light*. Elsevier.

[bb6] Glatter, O. & Kratky, O. (1982). *Small Angle X-ray Scattering*. Academic Press.

[bb7] Guilbaud, J. B. & Saiani, A. (2011). *Chem. Soc. Rev.* **40**, 1200–1210. 10.1039/c0cs00105h 21113529

[bb8] Guinier, A. (1963). *X-ray Diffraction in Crystals, Imperfect Crystals, and Amorphous Bodies.* Dover.

[bb9] Koch, M. H. J., Vachette, P. & Svergun, D. I. (2003). *Q. Rev. Biophys.* **36**, 147–227. 10.1017/s0033583503003871 14686102

[bb10] Liu, J., Costantino, I., Venugopalan, N., Fischetti, R. F., Hyman, B. T., Frosch, M. P., Gomez-Isla, T. & Makowski, L. (2016). *Sci. Rep.* **6**, 33079. 10.1038/srep33079 PMC5024092 27629394

[bb11] Liu, J. & Makowski, L. (2022). *Curr. Opin. Struct. Biol.* **75**, 102421. 10.1016/j.sbi.2022.102421 PMC11317818 35834949

[bb12] Liu, J., Connors, T., Hyman, B. T., Burghammer, M., Cotte, M. & Makowski, L. (2024). *Molecular Polymorphism of Tau Aggregates in Pick’s Disease.* Submitted. 10.1016/j.nbd.2025.107104 PMC12645749 40957494

[bb101] Mandelbrot, B. B. (1983). *The Fractal Geometry of Nature*, revised and enlarged ed. W. H. Freeman and Co.

[bb13] Martin, J. E. & Ackerson, B. J. (1985). *Phys. Rev. A*, **31**, 1180–1182. 10.1103/physreva.31.1180 9895605

[bb14] Martin, J. E. & Hurd, A. J. (1987). *J. Appl. Cryst.* **20**, 61–78.

[bb15] Nepal, P., Al Bashit, A., Yang, L. & Makowski, L. (2022). *J. Appl. Cryst.* **55**, 1562–1571. 10.1107/S1600576722009955 PMC9721334 36570653

[bb16] Nepal, P. & Saldin, D. K. (2018). *Phys. Rev. B*, **97**, 195426.

[bb17] Ogawa, T., Miyashita, S., Miyaji, H., Suehiro, S. & Hayashi, H. (1989). *J. Chem. Phys.* **90**, 2063–2067.

[bb18] Pfeifer, P. & Avnir, D. (1983). *J. Chem. Phys.* **79**, 3558–3565.

[bb19] Putnam, C. D., Hammel, M., Hura, G. L. & Tainer, J. A. (2007). *Q. Rev. Biophys.* **40**, 191–285. 10.1017/S0033583507004635 18078545

[bb20] Roe, R.-J. (2000). *Methods of X-ray and Neutron Scattering in Polymer Science.* Oxford University Press.

[bb21] Roig-Solvas, B. & Makowski, L. (2017). *J. Struct. Biol.* **200**, 248–257. 10.1016/j.jsb.2017.05.003 28511991

[bb22] Suzuki, T., Chiba, A. & Yarno, T. (1997). *Carbohydr. Polym.* **34**, 357–363.

[bb24] Svergun, D. I. & Koch, M. H. (2003). *Rep. Prog. Phys.* **66**, 1735–1782.

[bb23] Svergun, D. I. & Stuhrmann, H. B. (1991). *Acta Cryst.* A**47**, 736–744.

[bb25] Werner, M., Chott, A., Fabiano, A. & Battifora, H. (2000). *Am. J. Surg. Pathol.* **24**, 1016–1019. 10.1097/00000478-200007000-00014 10895825

[bb26] Yang, L. (2013). *J. Synchrotron Rad.* **20**, 211–218. 10.1107/S0909049512048984 23412476

[bb27] Yang, L., Antonelli, S., Chodankar, S., Byrnes, J., Lazo, E. & Qian, K. (2020). *J. Synchrotron Rad.* **27**, 804–812. 10.1107/S1600577520002362 PMC7206542 32381785

[bb28] Yang, L., Liu, J., Chodankar, S., Antonelli, S. & DiFabio, J. (2022). *J. Synchrotron Rad.* **29**, 540–548. 10.1107/S1600577521013266 PMC8900859 35254319

[bb29] Zohdi, V., Whelan, D. R., Wood, B. R., Pearson, J. T., Bambery, K. R. & Black, M. J. (2015). *PLoS One*, **10**, e0116491. 10.1371/journal.pone.0116491 PMC4339720 25710811

